# Advances in Anti-IgE Therapy

**DOI:** 10.1155/2015/317465

**Published:** 2015-05-05

**Authors:** Arzu Didem Yalcin

**Affiliations:** ^1^Internal Medicine, Allergy and Clinical Immunology, Near East University, Northern Cyprus, Mersin 10, Turkey; ^2^Genomics Research Center, Academia Sinica, Taipei, Taiwan; ^3^Antalya Education Research Hospital, Antalya, Turkey

## Abstract

Omalizumab depletes free IgE in the blood and interstitial space and inhibits IgE binding to Fc*ε*RI on basophils, mast cells, and dendritic cells. We stopped omalizumab treatment after four years. Recurrences of urticaria symptoms were found to be higher in patients with chronic urticaria than recurrences of asthmatic symptoms in severe persistent asthma patients. For the very first time, we used omalizumab in symptomatic therapy of recurrent laryngeal oedema and urticaria attacks in a patient with postoperative pulmonary carcinoid tumor for eight months. During the four years of follow-up, no recurrence was noted in pulmonary carcinoid tumor. Control PET CT results revealed normal findings. After omalizumab treatment, laryngeal oedema and urticaria symptoms were decreased. The most common adverse reaction from omalizumab is injection site induration, injection site itching, injection site pain, and bruising but the package insert contains warnings regarding parasitic infections. While there are no reports of fatal anaphylaxis as a result of omalizumab, some cases have been serious and potentially life-threatening. Therefore, the FDA requires that people receiving omalizumab be monitored in the physician's office for a period of time after their injections.

## 1. Introduction

Omalizumab, a humanized mAb that binds to the CH3 domain, near the binding site for the high-affinity type-I IgE Fc receptors of human IgE, can neutralize free IgE and inhibit the IgE allergic pathway without sensitizing mast cell and basophils. Omalizumab is a humanized recombinant anti-IgE monoclonal antibody approved for therapeutic use both in adults and in children aged 6–12 years with severe allergic asthma. The coexistence of severe asthma refractory to the conventional pharmacological approach and sensitization to at least one perennial allergen represent the current indications for omalizumab prescription. Its efficacy and safety as an add-on therapy is sustained by several data coming from both clinical trials and real life experiences [1–10] and showing a significant reduction of yearly exacerbation rate. Omalizumab, which has been conceptualized for treating IgE mediated allergic diseases and approved for treating patients with severe persistent allergic asthma in many countries, can neutralize IgE, impede the IgE allergic pathway, and render mast cells and basophils insensitive to activation through IgE/Fc*ε*RI. In addition to asthma, omalizumab has been investigated in various other conditions including chronic urticaria (CU), perennial and seasonal allergic rhinitis (AR), pruritic bullous pemphigoid, latex allergy, peanut allergy, idiopathic anaphylaxis, hyper-IgE syndrome, chronic rhinosinusitis, interstitial cystitis, aspirin sensitivity, mastocytosis, eosinophilic gastroenteritis, and atopic dermatitis. Most patients with chronic urticaria have an autoimmune cause: some patients produce IgE autoantibodies against autoantigens, such as thyroperoxidase or double stranded DNA, whereas other patients make IgG autoantibodies against Fc*ε*RI, IgE, or both, which might chronically activate mast cells and basophils. In the remainder of patients with CSU, the nature of the abnormalities has not yet been identified. Accumulating evidence has shown that IgE, by binding to Fc*ε*RI on mast cells without Fc*ε*RI cross-linking, can promote the proliferation and survival of mast cells and thus maintain and expand the pool of mast cells. IgE and Fc*ε*RI engagement can also decrease the release threshold of mast cells and increase their sensitivity to various stimuli through either Fc*ε*RI or other receptors for the degranulation process [11, 12]. The development of omalizumab therapy over the past 20 years provides an interesting example of the emergence of a conceptually new, biotechnology-produced pharmaceutical [13–18].

## 2. Omalizumab and IgE Receptors

In a patient with an allergic disease caused by type I hypersensitivity toward specific external antigens, omalizumab induces multifactorial therapeutic effects. Omalizumab depletes free IgE in the blood and interstitial space and inhibits IgE binding to Fc*ε*RI on basophils, mast cells, and dendritic cells. Omalizumab cannot bind to IgE that is already bound to Fc*ε*RI and does not have a direct effect on Fc*ε*RI levels. However, the depletion of free IgE results in the downregulation of Fc*ε*RI on cells bearing the receptor, making those cells insensitive to the stimulation by incoming allergens [16–19].

Omalizumab has been approved in over 120 countries for treating patients with SPA. These pharmaceutical developments have validated the IgE pathway as an effective therapeutic target for treating IgE mediated allergic disease [20–22].

## 3. Omalizumab Effects on sApo-2L and Allergen Specific Immunotherapy

Tumor necrosis factor related apoptosis inducing ligand (TRAIL: Apo-2L) is used as a marker for apoptosis. TRAIL (Apo-2L) is a transmembrane (type II) glycoprotein belonging to the TNF superfamily. The extracellular domain of TRAIL is homologous to that of other family members and shows a homotrimeric subunit structure. Like TNF and FasL, sApo-2L also exists physiologically in a biologically active soluble homotrimeric form. An increase in eosinophil levels has been reported in allergic asthma and is thought to reflect an increase in peripheral blood eosinophil survival promoted by Apo-2L.

In our previous study we showed that soluble Apo-2L levels in patients with severe persistent allergic asthma decreased after anti-IgE treatment using omalizumab. These results suggested that sApo-2L may act as a soluble effector molecule and that the decrease in levels after omalizumab treatment may allow us to use this marker to monitor clinical improvement. Combination therapy with omalizumab and specific subcutaneous immunotherapy (SCIT) in patients with severe persistent asthma also suggest that omalizumab is an effective therapy in such individuals. Omalizumab reduces serum IgE levels and Fc*ε*RI receptor expression on key cells in the inflammatory cascade. The consequences of these processes are the inhibition of the release of inflammatory mediators from mast cells and diminished recruitment of inflammatory cells, especially eosinophils, into the airways [22, 23].

Allergen specific immunotherapy (SIT) has the advantage of being the only causal treatment of allergic controlled asthma and rhinitis but is fraught with the dangers of severe systemic or local side effects and anaphylaxis. Omalizumab can possibly overcome these limitations by binding exclusively to circulating IgE molecules and reducing the levels of circulating IgE regardless of allergen specificity by binding to the constant region of circulating IgE molecule. This prevents free IgE from interacting with the high- and low-affinity IgE receptors (Fc′′RI and Fc′′RII) on mast cells, basophils, macrophages, dendritic cells, B lymphocytes and subsequently leads to a decrease in the release of the mediators of the IgE mediated allergic response, namely, cytokines, histamines, and leukotrienes [22, 24, 25].

The first clinical trial looking for the clinical effects of a combined therapy of SIT and omalizumab was performed in grass and birch pollen allergic children and adolescents in Germany. Kuehr and colleagues recruited 221 children and adolescents to evaluate the efficacy and safety of omalizumab with SIT on birch pollen induced allergic rhinitis (AR) [26]. SIT plus omalizumab-treated subjects were reported to have a 48% reduction in allergen-induced symptom load over two pollen seasons independent of the allergen. Furthermore, rescue medication use, number of days with symptoms, and symptom severity were significantly lower in the SIT plus omalizumab groups compared with SIT alone. A post hoc subanalysis of this study to assess the effects of each treatment (SIT or omalizumab) demonstrated that SIT alone did not significantly reduce either symptoms severity score [27]. Hence, combination therapy may be complimentary, providing the superior effect compared to individual treatments. Recently, there have been trials of omalizumab and SIT in patients with AR and comorbid asthma. In the trial by Kopp and colleagues, a significant reduction of 40% in symptom load was observed in favor of SIT plus omalizumab compared with SIT alone (*P* = 0.04) [28]. Another study showed that the tolerability of SIT after pretreatment with omalizumab or placebo in patients with symptomatic asthma was not adequately controlled with inhaled corticosteroids. A total of 13.5% of patients treated with omalizumab showed systemic allergic reactions to SIT compared to 27% in those receiving placebo (*P* = 0.017). More patients were able to reach the target maintenance SIT dose (*P* = 0.004) in the omalizumab group compared to placebo [29, 30], suggesting that pretreatment with omalizumab was associated with fewer systemic allergic reactions to SIT and enabled more patients to achieve the target immunotherapy maintenance dose [22, 31, 32].

## 4. Omalizumab Effects on Oxidative Stress Markers, Vitamin-D, and Homocysteine

Ceruloplasmin (CP) is a copper-containing alpha-2-glycoprotein with a molecular weight of approximately 132 kDa. Ceruloplasmin is essential for iron homeostasis, is involved in angiogenesis, and under different conditions can act as either a pro- or antioxidant. The known functions of ceruloplasmin oxidase activity (COA) include copper transportation, iron metabolism, antioxidant defense and involvement in angiogenesis, and coagulation. It was previously reported that synthesis of CP was stimulated by interleukin-1 in normal and copper deficient rat models concluding that CP was dependent on oxidase activity [33]. Moreover, the copper ions had been suggested as an explanation for the sensitivity of asthmatic individuals by their biologic effects of inhaled particulate air pollution.* In vivo* experiments on finding the cytokines involved in acute-phase protein response showed that there were three major cytokines: interleukin-1-beta, interleukin-6, and TNF-alpha [26, 34].

An imbalance between oxidative stress and antioxidative capacity may play an important role in the development and progression of bronchial asthma (BA) and chronic obstructive pulmonary disease (COPD). The systemic oxidant-antioxidant status changes during exacerbation versus stable periods in patients with BA and COPD. During an exacerbation period of BA, despite the decreases in glutathione peroxidase (GSH-Px), glutathione reductase (GRd) and melatonin levels, malondialdehyde (MDA) and catalase (CAT) levels, and the white blood cell count, the percentage of eosinophils is significantly higher than in the stable period. MDA and superoxide dismutase (SOD) values are higher in the exacerbation period than in the stable period although GSH-Px, GRd, melatonin, pH, and pO_2_ values are lower in the exacerbation period than in the stable period. Blood counts and respiratory function tests were reported not to change between exacerbation and stable periods in patients with COPD. Thus episodes of BA or COPD might be associated with elevated levels of oxidative stress.

A decrease in NO during omalizumab therapy was also previously described by Barber and Cousins [34]. Downregulation of ET-1 in EBC significantly correlates with a decrease in the markers of allergic (and eosinophilic) inflammation, such as NO, ECP, or blood eosinophil counts, as well as increase in spirometric indices. These changes were observed after 16 weeks of therapy. A follow-up observation performed after 52 weeks of treatment revealed a further significant fall in ET-1 concentrations in EBC; however, the improvement of other markers of allergic inflammation was less pronounced. This could indicate that anti-IgE therapy has its greatest influence on eosinophilic inflammation during the first 16 weeks of therapy. Nevertheless the effects of many other immunological mechanisms related to remodeling, as well as the known action and interactions of ET-1 observed in the first period of treatment, are thought to continue over time. This suggests that longer-term anti-IgE therapy with omalizumab in asthmatic patients could significantly limit the development of inflammation and bronchial structural changes. In our previous study we investigated changes in total antioxidant capacity in asthmatic patients treated with omalizumab. Our data suggested that ongoing therapy with omalizumab is already proven to be clinically effective in treatment of severe allergic asthma. Anti-IgE therapy is an innovative and promising treatment modality that mediates its effects in part at least through decreased inflammation following improved antioxidant capability. In turn, our study was suggesting that measuring of the latter may prove to be useful surrogate markers to monitor efficacy of treatment in patients suffering from this disease [22].

Alternatively, the development of atopy may also be a direct effect of elevated homocysteine or some of its metabolites, which appears to exert a number of diverse effects on immune function. In addition, total homocysteine (Hcy) has been shown to increase in response to immune activation and cell proliferation during a nonallergic Th1-type immune response. Although much less is known about the health effects of sustained postload homocysteine concentrations, there is evidence that it has negative effects on platelet aggregation and endothelial function. A number of studies have indicated that homocysteine may contribute to the development and progression of atherosclerosis, a risk factor for cardiovascular diseases. However, the mechanisms by which Hcy can induce vascular dysfunction are not fully understood [35–41].

Vitamin-D (25(OH)D) has effects on the innate and adaptive immune system. 25(OH)D levels are associated with poor asthma control, reduced pulmonary function, increased medication intake, and exacerbations. Little is known about 25(OH)D in adult asthma patients or its association with asthma severity [42, 43]. More than that, 25(OH)D triggers a Hcy metabolizing enzyme and data from the Longitudinal Aging Study Amsterdam suggested a correlation between 25(OH)D status and Hcy levels [44]. The decrease in Hcy concentrations and increase in 25(OH)D also support the possible vascular endothelial protection mechanism.

## 5. Omalizumab Effects on Pruritic Bullous Pemphigoid

Bullous pemphigoid (BP) is an acquired, autoimmune, bullous disease that is characterized by autoantibodies against the 230-kDa bullous pemphigoid antigen within basal keratinocytes and the 180-kDa type XVII collagen within the basement membrane zone lying between the epidermis and dermis. In addition to skin blisters, patients with BP often experience pruritus and erythematous urticaria-like skin lesions.

CD200 (OX-2) is a novel immune-effective molecule, both cell membrane-bound and also existing in a soluble form in serum (sCD200, sOX-2), act as a proinflamatory through its receptor [11, 14, 22]. In our previous study, we reported a patient who had a pruritic bullous pemphigoid and very high levels of total IgE (5000 kU/L) who was refractory to the aggressive immunosuppressive regimens for bullous pemphigoid but responded rapidly to systemic anti-IgE. The circulating level of sOX-2 was 48.45 pg/mL in serum and 243 pg/mL in blister fluid. Soluble OX-2 levels were higher in blister fluid than in serum. During the second month of follow-up, the patient's sOX-2 level decreased to 26.7 pg/mL. Clinical improvement was demonstrated as histological reepithelialization. Optimal treatment modalities need to be clarified in such situations. After the second round of omalizumab (300 mg), frequency of exacerbations decreased and after 13th round it was completely disappeared [45]. Reduction in serum levels sOX-2 with anti-IgE treatment suggests that sOX-2 could be proinflammatory [21–23, 45]. Soluble OX-2 might also play a role in immune response in the pathogenesis of autoimmune and inflammatory skin disorders [45].

## 6. Omalizumab Effects on Coagulation Pathway

More interestingly, as in some of our cases we have observed, one with severe persistent asthma (SPA) patient who had protein C/S deficiency history, multiple massive pulmonary embolus, and systemic subacute thrombosis determined in vena saphena parva and in left vena perforantes cruris underwent omalizumab treatment. After a long term (20 months) treatment with omalizumab, he had a decreased fractional exhaled nitric oxide concentrations (FENO), d-dimer (DD), sTRAIL, proinflammatory IL-1*β*, and OX-2 and had an increased CXCL8, activated pC (APC), antithrombin III (AIII), protein S (pS), and protein C (pC) levels [11]. In this patient's blood levels of APC, AIII, pS, and pC were found to be increased (74, 128%, 102%, and 86%, resp.), and DD level (412 U/L) was found to be decreased at the 30th month under omalizumab therapy and this results were significant [46].

Severe persistent asthma is associated with a procoagulant state in the bronchoalveolar space and is further aggravated by impaired local activities of the anticoagulant pC/S, AIII system, and fibrinolysis, as demonstrated by massive fibrin depositions in the alveoli of a SPA who died from a SPA attack who did not respond to treatment. Recent reports revealed that patients with CU also show signs of thrombin generation and activation of the TF pathway of the coagulation system. DD, a fibrin degradation product formed during the lysis of a thrombus, is also detected in high levels in patients with active CU [47–51]. After omalizumab therapy, significant decrease of the levels of DD shows the importance of procoagulant state in allergic patients. We also believe that DD may also have an important role for the relationship between IgE and extrinsic coagulation pathway in the endothelial cells [46]. The biologic effects of APC and pC can be divided into anticoagulant and cytoprotective effects. [48] In patients with SPA bronchoalveolar levels of APC decreased after a bronchial allergen challenge and were significantly lower than healthy controls and APC/pC ratios were decreased in induced sputum of patients with SPA, pointing to an imbalance between coagulation and the pC system [49, 50]. We think that omalizumab inhibited activation of extrinsic coagulation pathway and lowered d-dimer level by blocking free IgE. Because of this, we think that omalizumab has a similar effect with heparin. After the injection of heparin, an increase in the percentage of protein C/S has been observed. Anticoagulant treatment with heparin and warfarin had been attempted to reduce the symptoms of CU and SPA; however inhaled heparin is no longer used in clinical practice as adjunctive therapy for SPA attacks because of equivocal results [50–53].

**Figure 1:**
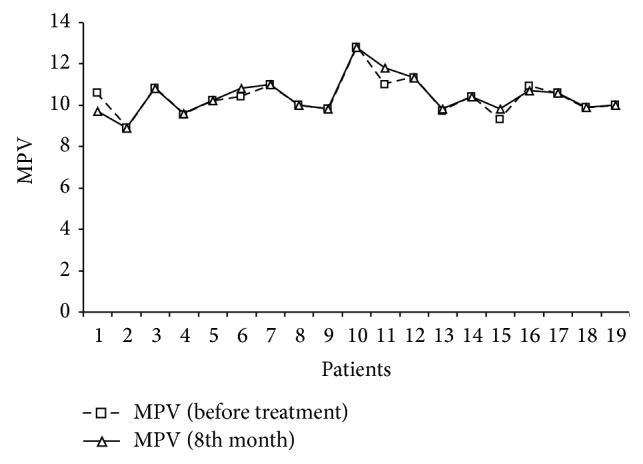
The insignificant MPV level difference was observed between before-omalizumab and after omalizumab period.

**Figure 2:**
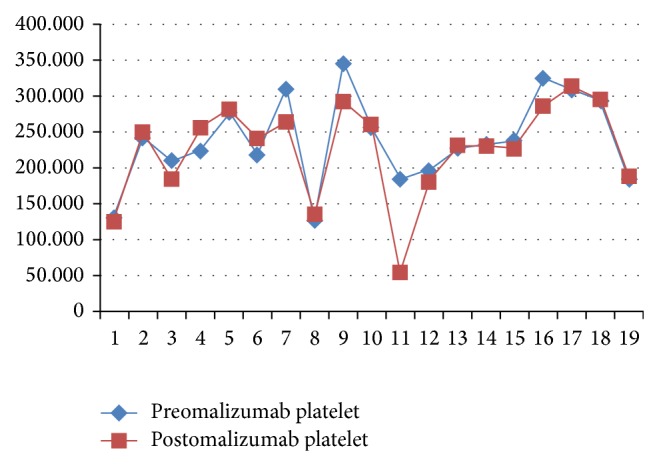
The insignificant platelet level difference was observed between before-omalizumab and after omalizumab period.

**Figure 3:**
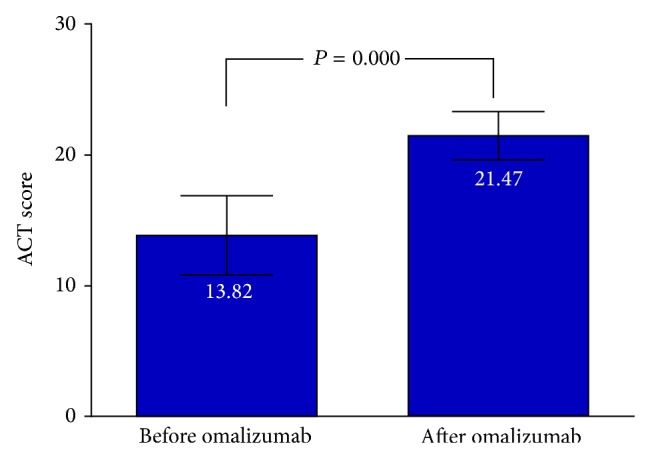
Asthma control test asthma control test (ACT) (Quality Metric Inc.) score of <20, indicating that asthma was not well controlled (before omalizumab therapy and a year later).

**Figure 4:**
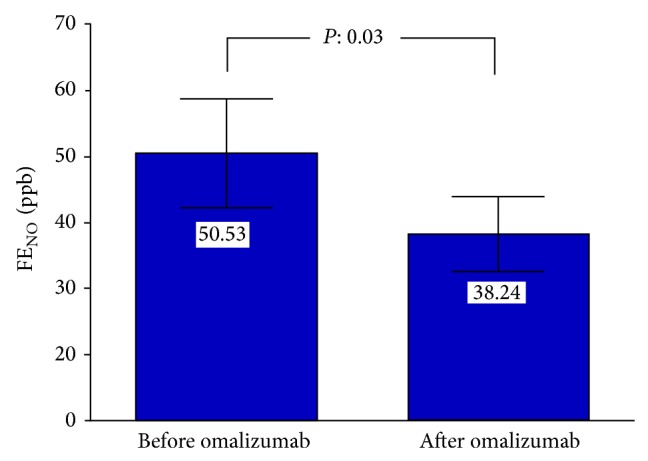
Fractional exhale nitric oxide concentrations (FENO) (ppb) (before omalizumab therapy and a year later).

The function of platelets is well known in haemostasis but also platelets are fully functional cells concurrently with haemostasis. Previous studies suggested that platelets have a role in asthma pathogenesis in development of bronchoconstriction, airway inflammation, airway remodelling, and bronchial hyperresponsiveness. Lifestyle modification, antihypertensive, lipid lowering, and diet therapies can affect MPV values, but these effects need to be investigated with thrombotic endpoints. It was previously suggested that increased MPV values are predictors of early atherosclerosis. However, there were conflicting results in the association of asthma and atherosclerosis. And if MPV value is an indicator of inflammation and atherosclerosis, increased MPV values may be associated with asthma. However, we could not find any difference in MPV values of patients both in pre- and postomalizumab period. Thrombocytopenia developed in one male patient (number 11) after the 22nd dose of the drug was given. When the platelet count fell down to 55,000/mm^3^, the omalizumab treatment was suspended for 4 weeks until the platelet count rose up to 100,000/mm^3^  ([20], Figures 1, 2, 3, and 4).

## 7. Omalizumab Effects on Hyperimmunoglobulin-E Syndrome, Eosinophilic Gastroenteritis, Mastocytosis

Hyperimmunoglobulin E syndrome (HIES) is a heterogeneous group of immune disorders. It is characterized by very high concentrations of the serum antibody IgE. Clinically eczema-like rash, cold staphylococcal infection, severe lung infection are seen. An IgE level greater than 2,000 IU/mL is often considered diagnostic, except patients younger than 6 months of age. Extrinsic pathway of coagulation is activated in response to high level of circulatory IgE. Abnormal neutrophil chemotaxis due to decreased production of interferon gamma by T lymphocytes is thought to cause the disease. Both autosomal dominant and recessive inheritance have been described [54, 55]. Mutations in molecules DOCK8 have been associated with syndromes that share many features with classical autosomal dominant HIES, which is inherited by an autosomal recessive trait and tend to have a milder clinical picture [55, 56]. STAT3 is a key regulator of many immunologic pathways. It is involved in the signal transduction of many cytokines, including but not limited to IL-6, IL-10, IL-21, IL-22, and IL-23 [57]. Animals with a myeloid-specific deletion of STAT3 lead to upregulation of many Th1 cytokines, such as IFN*γ* and TNF*α*, and downregulation of proinflammatory and anti-inflammatory responses regulated by IL-6 and IL-10, respectively [55, 56]. These cytokines are critical to differentiation of TH17 cells, which are important in inflammatory response to bacterial and fungal pathogens. It was reported that both STAT3 mutation-positive and STAT3 mutation-negative HIES exhibited a profound deficit in TH17 differentiation. Several studies reported clinical improvement in patients with severe atopic eczema with high serum IgE level [58, 59].

Eosinophilic gastroenteritis (EGE) is characterized by patchy or diffuse eosinophilic infiltration of any part of gastrointestinal (GI) tract [60, 61]. Eosinophils are normally present in gastrointestinal mucosa, but deeper infiltration and more than 30 eosinophils per high-power field in at least five areas are pathologic [62]. Since GI tract is frequently faced with external allergens via ingested foods, allergens from food pass the mucosa and trigger an inflammatory response that lead mast cell degranulation and recruit eosinophils. Tissue damage is caused by cytotoxic proteins contained in the cytoplasmic granules of eosinophil. In addition to tissue eosinophil, eosinophil can also mediate proinflammatory effects, that is, upregulation of adhesion systems, modulation of cell trafficking, releasing chemokines (eotaxin), lipid mediators, and leukotriene. Eosinophil recruitment into the tissue is regulated by a number of inflammatory cytokines, that is, IL-3, IL-4, IL-5, IL-13, granulocyte macrophage colony stimulating factor (GM-CSF), and T helper 2 (Th2) cytokines. A Th2-type immune response seems to be involved in both IgE and non-IgE mediated EGE [63]. Anti-IgE treatment with omalizumab is associated with a 35–45% drop in peripheral blood eosinophil count as well as decrease in duodenal and antral eosinophil count [64, 65]. It also effectively blocks CD23 mediated allergen binding to B cells. But some reports failed to demonstrate* in vivo* immunomodulatory activity on T cell responses [66].

Mastocytosis is a heterogeneous disorder that results from clonal mast cell proliferation (myeloproliferative neoplasm), characterized by excessive mast cell accumulation in skin (cutaneous form) or multiple tissues, with or without skin involvement (systemic form) [67, 68]. Increased local concentration of soluble mast cell growth factors in lesions are believed to stimulate mast cell proliferation. Impaired mast cell apoptosis and interleukin-6 have also been postulated to be involved, as evidenced by BCL-2 upregulation and IL-6 elevation in tissue. Some activating point mutation of c-kit in codon 816 (usually KITD816V), encoding the tyrosine kinase receptor for stem cell factor, are found to be associated with systemic form. Since omalizumab reduces the expression of Fc*ε*RI on circulating basophils and mast cells, it seems to lower the activity potentials of basophils and mast cells, thereby reducing the potential reactivity of these cells [65, 69]. Concordantly, serum tryptase was reported to decrease under omalizumab therapy in two mastocytosis patients, but it remained unchanged in two other patients [69–81].

## 8. Omalizumab Effects on Nasal Polyps and Samter's Syndromes

The historic triad of nasal polyposis, asthma, and intolerance to aspirin and related chemicals, recently designated as Samter's syndrome, is an inflammatory condition of unknown pathogenesis. Many patients with Samter's syndrome also have a marked eosinophilia of both bronchial and nasal secretions as well as the circulating blood. Approximately 10% of the patients have urticaria-angioedema, alone or in combination with respiratory inflammation. As with all allergic diseases, the cornerstone of treatment is environmental control with avoidance of respiratory irritants, aspirin, and aspirin-like medications. Management of upper airway disease requires careful prescription of medication supplemented by judicious selection of surgery. Omalizumab demonstrated clinical efficacy in the treatment of nasal polyps with comorbid asthma, supporting the importance and functionality of local IgE formation in the airways [76], but in our study, no change was seen on nasal polyposis after omalizumab treatment [21]. Nasal polyps from patients with Samter's triad had a significantly higher inducible nitric oxide synthase activity when compared with the nasal polyp patients without Samter's syndrome [77].

## 9. Omalizumab Effects on Atopic Dermatitis

Severe refractory atopic dermatitis is a chronic, debilitating condition that is associated with elevated serum IgE levels. The mechanisms of omalizumab in the treatment of atopic dermatitis (AD) need further research in lowering serum IgE subjects. Several case reports investigating anti-IgE therapy in patients with AD found symptomatic improvement with omalizumab [13]. Recently, Ozdemir et al. [68] showed that all patients receiving omalizumab had strikingly decreased levels of TSLP, OX40L, TARC (involved in Th2 polarization), and interleukin-9 compared to placebo in their randomized, placebo-controlled clinical trial. In addition, they found a marked increase in IL-10, a tolerogenic cytokine, in the omalizumab-treated group. Patients on anti-IgE therapy had an improvement in clinical outcomes.

## 10. Omalizumab Effects on Chronic Urticaria

Metz et al. [74] assessed responder rates, optimal dosage, response to up-/downdosing, time to relief of symptoms, rates of return and time of relapse after omalizumab administration, and safety in 51 CU patients, 20 with chronic spontaneous urticaria (CSU) alone, 21 with different forms of chronic inducible urticaria (CindU) and 10 with both in their clinical analysis. They showed that omalizumab was a rapidly acting, highly effective, and safe drug in CSU and CindU patients in their clinical experience from more than 1250 injections in those patients over four years. Their observations in a real life clinical setting support the recommendation of current EAACI/GA2LEN/EDF/WAO guideline for the management of urticaria to use omalizumab on the treatment of urticaria patients [75].

The activation of mast cells and their release of inflammatory mediators are regarded as the ‘‘final common pathway” for a myriad of proinflammatory factors, including those involved in the various types of urticaria [82, 83].

The clinical response of urticaria to H1-antihistamines and the finding of increased concentrations of histamine in skin tissue fluid underscore the role of histamine derived from dermal mast cells as a major mediator of urticaria. Although highly unlikely, it cannot be excluded that in some cases of urticaria, the primary abnormality lies in the mast cells themselves. If this were the case, it would be likely that the condition would be systemic rather than confined to the skin. Therefore it is more likely that skin mast cells in patients with urticaria are not intrinsically abnormal but become increasingly sensitive or ‘‘unstable” or activated as the result of certain abnormal factors present in their surroundings. Although there are many nonimmunologic factors that might influence mast cell function in the skin, such as components of the complement system and neuropeptides, particularly those related to stress, because this review is primarily concerned with the mechanisms by which omalizumab might be effective, nonimmunologic factors will not be considered in detail [84–89].

In conventional thinking the involvement of IgE in mast cell activation requires the cross-linking of Fc*ε*RI-bound IgE by antigen or anti-IgE antibodies. This initiates the aggregation of Fc*ε*RI, leading to tyrosine kinase activation and subsequent mast cell activation for secretion. However, in 2001, it was suggested independently by 2 groups that monomeric IgE in the absence of antigen can have multiple effects in murine mast cells, including differentiation, proliferation, survival, and mediator and cytokine generation. These effects, which involve the binding of IgE to Fc*ε*RI and the aggregation of Fc*ε*RI, occur without the mast cells undergoing degranulation. The finding that monomeric IgE can augment mast cell activity has been confirmed by studies using various techniques. In a transcriptome analysis of 8793 genes, sensitization of mast cells with monoclonal IgE alone, without Fc*ε*RI cross-linking, was found to upregulate 58 genes more than 2-fold compared with their levels in unsensitized mast cells. These genes included those for cytokines, such as IL-1b, IL-6, and colony stimulating factor 1; chemokines, such as CXCL8, CCL4, and CCL7; and cytokine and chemokine receptors. The genes for various immune regulators, adhesion molecules, antiapoptosis proteins, and cytoskeletal elements, such as RAS protein activator like 1 and fibronectin leucine-rich transmembrane protein 2, were also upregulated [89–91].

First, omalizumab sequesters monomeric IgE to reduce its priming effect on mast cells. This would be particularly relevant if HC IgE is involved in the pathogenesis of urticaria. Second, in patients with IgG autoantibodies against IgE or Fc*ε*RI, the depletion of mast cell-bound IgE by omalizumab and the subsequent downregulation of Fc*ε*RI on mast cells and basophils would lead to their decreased state of hyperexcitability. Third, in those patients with IgE autoantibodies against autoallergens, the inhibition of IgE binding to Fc*ε*RI by omalizumab and the downregulation of Fc*ε*RI would represent a central mechanism of omalizumab [84]. Other studies have followed the suggestion that mouse monoclonal IgE molecules are heterogeneous with respect to their ability to induce survival and activation events in mast cells [92].

We stopped omalizumab treatment after 4 years. Recurrences of urticaria symptoms were found to be higher in patients with chronic urticaria than recurrences of asthmatic symptoms in severe persistent asthma patients. This might be due to transient effect of omalizumab on extrinsic coagulation pathway in the endothelial cells.

## 11. Omalizumab and Cardiovascular Safety

There have been concerns about the cardiovascular safety in patients initiating omalizumab therapy, because of the most recent study that analyzed the association between omalizumab and arterial thrombotic events [79–81, 93]. We showed that, in one of our patient, Doppler ultrasonography did not reveal any thrombus after anti-IgE therapy and the patient did not require lung transplantation and that serum protein S/C levels increased to normal ranges. Exercise stress testing was normal and after initiation of anti-IgE treatment, neither cranial emboli event nor any neurologic complications did occur. Patient did not report any cardiac arrhythmias after initiation of anti-IgE therapy. Besides, exercise stress testing was normal, while the patient was treated with anti-IgE. Aneurysm enlargement or complications were not detected during the treatment with anti-IgE [23].

## 12. Omalizumab Effects on Diabetes Mellitus

The clinical experience during the patient follow-up of omalizumab-treated severe persistent allergic asthma patients with type-2 diabetes mellitus is introduced. Omalizumab is generally considered safe. The most common adverse reaction from omalizumab is injection site pain and bruising but the package insert contains warnings regarding malignancies, geohelminth infections, and a “black box” warning about anaphylaxis. While there are no reports of fatal anaphylaxis as a result of Xolair, some cases have been serious and potentially life-threatening. Therefore, the FDA requires that people receiving Xolair be monitored in the physician's office for a period of time after their injections. Also, it is not yet known what the potential long term effects of Xolair use may have on people who are prone to getting cancer (such as the elderly). While it would appear that Xolair has potentially severe side effects, it must be remembered that anaphylaxis and cancer formation occurred only in a very small number of patients. Moreover, our knowledge about omalizumab use for asthma and other allergic diseases has improved to such an extent that we now better understand the treatment influence on systemic levels of oxidative stress markers, the interaction of oxidant and antioxidant balance, and apoptotic and inflammatory markers. We showed that omalizumab therapy increases blood glucose levels in allergic asthma patients with diabetes mellitus. Although we do not know the exact mechanism behind this relationship, it might be related with vial containing (145 mg sucrose) of omalizumab. Patients with diabetes mellitus should be informed that such a need of insulin dose should be increased due to the possible effect of omalizumab on blood glucose level. In these two patients half of the recommended dosage was given and blood glucose levels were controlled [22].

## 13. There Might Be a Risk of Anaphylaxis due to Omalizumab Therapy

The most common adverse reaction from omalizumab is injection site induration, injection site itching, injection site pain, and bruising but the package insert contains warnings regarding parasitic infections. While there are no reports of fatal anaphylaxis as a result of omalizumab, some cases have been serious and potentially life-threatening. Therefore, the FDA requires that people receiving omalizumab be monitored in the physician's office for a period of time after their injections [20].

A female patient with severe persistent allergic asthma, aged 58, is introduced. Laboratory tests are as follows: anti-nuclear antibody, and viral hepatitis markers such as HBsAg, HBsAb, anti-HCV HIV, thyroid antibodies were negative, autologous serum skin test was positive. Liver, thyroid, and renal function tests, serum IgG, IgA, and IgM levels were within normal ranges. Skin prick tests (SPTs) were highly positive for mite and mold. The specific IgE levels were correlated with the SPTs. Total IgE level was 480 IU/L. Body temperature was 38.5°C.

The patient had not previously reported drug induced anaphylaxis. On omalizumab (on the 23rd dose) therapy, this patient had laryngeal oedema during a complicated common cold infection with bacterial sinusitis after omalizumab injection.

Systemic steroids (500 mg prednisolone), ketotifen fumarate (1X1), antibiotic and antihistamines (desloratadine, 1X1) were given. Oral antihistaminics and mast cell stabilizing agents were used for treatment afterwards. We did not stop omalizumab therapy. We do not suggest omalizumab during the first five days of complicated viral infections such as common cold and bacterial sinus infections [94].

## 14. Omalizumab and the Risk of Malignancy

I had a male patients with food allergy with pulmonary carcinoid tumor, aged 23. Autologous serum skin test was positive in patients. Anti-nuclear antibody and viral hepatitis markers such as HBsAg, HBsAb, anti HCV HIV, thyroid antibodies were negative in patients. Liver, thyroid, and renal function tests, serum IgG, IgA, and IgM levels were within normal ranges. Skin prick tests (SPTs) were highly positive for kiwi, tomato, fish, and orange. The specific IgE levels were correlated with the SPTs. Total IgE level was 960 IU/L (normal range: 0–100 IU/L). A mass was defined on lower lobe of left lung on computerized tomography (PET CT SUVmax: 6). The patient was operated. In postoperative period, he had recurrent laryngeal oedema and urticaria attacks. Omalizumab treatment planned because of the patient was resistant to antihistaminics and steroids. For the very first time, we used omalizumab in symptomatic therapy of recurrent laryngeal oedema and urticaria attacks in a patient with postoperative pulmonary carcinoid tumor for eight months. During the four years of follow-up, no recurrence was noted in carcinoid tumor. Control PET CT and CT results revealed normal findings. After omalizumab treatment, laryngeal oedema and urticaria symptoms were decreased. Oral antihistaminics and mast cell stabilizing drugs were used for treatment afterwards. Oral steroid was given only once [95].

Omalizumab has the potential to be an additional and solitary treatment option in patients with allergic bronchopulmonary aspergillosis and cystic fibrosis. Early onset treatment may be beneficial and patients with early stage of lung disease seem to benefit more [96]. Pooled data analysis revealed that a causal relationship between omalizumab therapy and malignancy is unlikely [97].

To sum up what I would like to express as a conclusion is that omalizumab in patients with severe persistent asthma is an effective therapy for asthma and comorbid conditions (CU, bee venom allergy, latex allergy, multidrug allergy, atopic dermatitis, food allergy, and Samter's syndrome) just like I have mentioned above. The mechanism of action of omalizumab in the treatment of asthma is believed to be multifactorial and includes effects mediated through altered production of redox metabolites, oxidative markers related mi RNA, TRAIL related mi RNA, and regulation of production of known inflammatory proteins.
